# A retrospective observational analysis of red blood cell transfusion practices in stable, non-bleeding adult patients admitted to nine medical-surgical intensive care units

**DOI:** 10.1186/s40560-019-0375-3

**Published:** 2019-04-04

**Authors:** Lesley J. J. Soril, Tom W. Noseworthy, Henry T. Stelfox, David A. Zygun, Fiona M. Clement

**Affiliations:** 1 Department of Community Health Sciences, Cumming School of Medicine, University of Calgary, 3D18, Teaching Research and Wellness Building, 3280 Hospital Drive NW, Calgary, Alberta T2N 4N1 Canada; 2 O’Brien Institute for Public Health, University of Calgary, 3280 Hospital Drive NW, Calgary, Alberta T2N 4N1 Canada; 3 Department of Critical Care Medicine, Cumming School of Medicine, University of Calgary, Foothills Medical Centre, 1403 29 Street NW, Calgary, Alberta T2N 2T9 Canada; 4 Department of Critical Care Medicine, Alberta Health Services and Faculty of Medicine and Dentistry, University of Alberta, Room 2-124 Clinical Sciences Building, 8440 - 112 Street, Edmonton, Alberta T6G 2B7 Canada

**Keywords:** Red blood cells, Blood transfusion, Restrictive transfusion threshold, Liberal transfusion threshold, Intensive care unit, Overuse

## Abstract

**Background:**

Red blood cell (RBC) transfusions are common procedures performed in the intensive care unit (ICU). However, conservative transfusion approaches have been recommended to avoid RBC transfusions that are not clinically necessary and to achieve optimal patient outcomes. The objective of this study was to examine the utilization and costs of RBC transfusions in medical-surgical ICUs and to compare this information against clinical guideline recommendations for best practice.

**Methods:**

Retrospective observational analysis of RBC transfusions in stable, non-bleeding adult patients was examined in a geographically-defined, population-based cohort of nine integrated ICUs between April 1, 2014 and December 31, 2016. RBC transfusions associated with a pre-transfusion hemoglobin value of 70 g/L or more were examined through linear and logistic regression. The total costs of RBC transfusions, based on the RBC unit cost, were estimated.

**Results:**

A total of 4632 RBC transfusions (2287 ICU admissions) were included. Pre-transfusion hemoglobin values were identified for 4487 transfusions. On average, 61% occurred at or above a hemoglobin value of 70 g/L (mean 73.4 ± 9.2 g/L). Factors associated with such transfusions included being male, age over 75, Sequential Organ Failure Assessment (SOFA) score greater or equal to 10, transfer from operating room, gastrointestinal bleeding, and trauma. A pre-transfusion hemoglobin value at or above 70 g/L was associated with increased odds of ICU mortality; there was no impact on overall hospital mortality. The total estimated cost of RBC transfusions was $2.99M Canadian dollars (CAD), with $1.82M CAD attributed to those with a hemoglobin value at or above 70 g/L.

**Conclusions:**

Over half of the examined RBC transfusions may not have aligned with recommended best practice; this suggests significant opportunity for improvement. The present findings are an essential step towards optimizing RBC transfusions in the ICU.

**Electronic supplementary material:**

The online version of this article (10.1186/s40560-019-0375-3) contains supplementary material, which is available to authorized users.

## Background

The need to address medical overuse, defined as “the provision of medical services for which the potential for harm exceeds the potential for benefit” [1], is increasingly being recognized by healthcare systems internationally [2–5]. In 2012, the Joint Commission on medical overuse in the USA identified a list of the top five overused medical procedures based on available evidence and expert-recommended standards of appropriateness [6]; among those listed was the over-transfusion of blood and blood products, such as red blood cells (RBCs).

Allogeneic RBC transfusion is a common procedure for many medical and surgical specialties and is typically used to manage hemorrhagic or anemic events among hospitalized patients [7–11]. However, with the risks of infection or even mortality associated with RBC transfusions, overuse is of significant concern for patient safety and quality of care [12, 13]. Beyond this, blood products are costly resources and are limited in their availability; current estimates suggest that acquisition and administration costs for RBC transfusions range internationally from $500 to $1200 USD per RBC unit transfused [14]. Blood conservation is, therefore, additionally important to maintaining financial stewardship in healthcare systems worldwide.

To guide appropriate use of RBC transfusion, clinical guidelines such as those from the AABB (formerly the American Association of Blood Banks) [15] have developed evidence-based recommendations predicated on set pre-transfusion hemoglobin concentration values. For example, for most stable, non-bleeding hospitalized patients, including those admitted to the intensive care unit (ICU), RBC transfusions are not recommended above a hemoglobin level of 70 g/L [15]. Informing such recommendations are a number of randomized controlled trials (RCTs) that have examined the efficacy of a restrictive (e.g., hemoglobin value of 70 g/L) versus a liberal transfusion strategy (e.g., hemoglobin value of 100 g/L). [16–20]. A recent systematic review of 31 RCTs found that, with the exception of certain high-risk groups (i.e., neurological injury or disorders, acute coronary syndrome), a restrictive transfusion strategy decreased transfusion requirements without increasing the risk of mortality and adverse outcomes for most hospitalized patients [21].

Given this established evidence base, the primary objective of this retrospective observational study was to measure the utilization and costs of RBC transfusion practices among medical-surgical ICUs and to compare this information to recommended best practice.

## Methods

### Study design and setting

For this multi-center, retrospective observational study, all RBC transfusion events in nine adult ICUs in Alberta, Canada, were examined between April 1, 2014 and December 31, 2016. The included ICUs are mixed medical and surgical units situated within academic, tertiary, and large urban or regional hospitals. Attending physicians (i.e., intensivists) are accountable for the medical care delivery in each ICU, with clinical coverage provided by post-graduate medical education trainees in most units [22]. Therefore, orders for laboratory tests and procedures, such as RBC transfusions, are made at the discretion of the attending intensivists or their delegates.

### Study population

Critically ill patients 18 years of age or older, who were (a) admitted to one of the nine adult ICUs and with a length-of-stay more than 24 h, (b) received at least one packed RBC transfusion during their ICU stay, and (c) captured in the *eCritical* database (see the “Data sources” section below), were included.

ICU patients were excluded for any of the following reasons: age less than 18 years, evidence of active blood loss, primary or secondary diagnosis of anemia, pregnancy, brain death or imminent death within 24 h, primary diagnosis of neurocritical illness, primary or secondary diagnosis of acute myocardial infarction, and admission after a routine cardiac surgical procedure. The definitions for each exclusion criterion are provided in Table 1, and complete operational definitions are provided in Additional file 1.

**Table 1:** Exclusion criteria for study cohort

Exclusion criteria	Definition(s)
Not an adult	Age less than 18 years at time of ICU admission
Active blood loss prior to transfusion	Decrease in Hgb of 30 g/L 12 h before the index transfusion
	3 units (900 mL) of RBC 12 h before the index transfusion
	One or more transfusions 12 h after the index transfusion
	Other indicators of active blood loss
Anemia	Primary or secondary diagnosis of anemia
Pregnancy	Pregnant state during in-hospital stay
Brain death or imminent death	Indication of clinical brain death in ICU or death within 24 h of ICU admission
Acute myocardial infarction (AMI)	Primary or secondary diagnosis of AMI
Neurocritical illness	Critically ill patients with neurological injury or condition of the brain requiring intensive care treatment (e.g., traumatic brain injury, intracerebral, or subarachnoid hemorrhage)
ICU admission after routine cardiac surgery	ICU admission of post-operative cardiac patients (e.g., coronary artery bypass grafting)

### Data sources

The primary data source was the clinical information system *eCritical Alberta* [23]. *eCritical* captures comprehensive data on each ICU admission and enables electronic documentation of patient information at the bedside. *eCritical* is also linked to an integrated clinical analytics and data warehouse system for all ICU patient data in the province [23]. Specifically for this study, the information obtained from *eCritical* included patient demographics (e.g., age, sex); diagnostic or case-mix (e.g., primary ICU admission diagnoses, secondary chronic conditions, Acute Physiology and Chronic Health Evaluation [APACHE] II, and Sequential Organ Failure Assessment [SOFA] scores); treatment data (e.g., RBC transfusions); laboratory data (e.g., hemoglobin measurements); and outcomes (e.g., ICU length of stay [LOS] and mortality). The start of the study period aligns to the commencement of data collection from all of the nine ICUs included in the *eCritical* system.

A secondary data source, the Canadian Institute for Health Information (CIHI) Discharge Abstract Database (DAD), which collects standardized administrative aspects of in-hospital care [24], was used to analyze information on hospital LOS, Charlson comorbidity index scores, 30-day and 60-day readmission to hospital, and in-hospital mortality. Each patient was assigned a unique personal identifier to permit linkage of patient information across databases.

### Primary and secondary outcomes

The primary outcome was the proportion of RBC transfusions associated with a pre-transfusion hemoglobin value greater than or equal to 70 g/L. The pre-transfusion hemoglobin value was defined as the most proximal hemoglobin measurement obtained within 24 h prior to the transfusion; this was assumed to be the threshold at which a transfusion was initiated. Secondary outcomes included ICU and hospital LOS and mortality.

### Data analysis

Patient and RBC transfusion characteristics were examined using descriptive statistics. Continuous data was presented as means and standard deviations (SD) or, if non-normal, as medians with interquartile ranges (IQR). The aggregate frequencies and percentages of RBC transfusion events within pre-transfusion hemoglobin ranges were depicted in tables and graphical form. For each month of the study period, the percentage of RBC transfusion events with a pre-transfusion hemoglobin value at or above 70 g/L was estimated and plotted with the 95% confidence interval (CI) over the 33 months. Simple linear regression was performed to examine the month-to-month change in the proportion of RBC transfusions associated with a pre-transfusion hemoglobin value above 70 g/L across the study period. In addition, multiple logistic and linear regression were performed to examine the association between (a) patient characteristics and the primary outcome and (b) pre-transfusion hemoglobin values of 70 g/L or more and the secondary outcomes. Clinically important covariates were incorporated into the final adjusted models and included: sex; age; APACHE II score; SOFA score; location prior to ICU admission (e.g., emergency department, operating room, or recovery room); ICU admit diagnostic category; Charlson comorbidity index score; mechanical ventilation status; and overnight timing of transfusion (i.e., between 19:00 and 7:00). Measures of effect estimated from logistic regression analyses were reported as unadjusted and adjusted odds ratios (OR) and 95% CI. Regression coefficients (*β*_1_), standard errors (SE), 95% CI, and *r*-squared values were reported from linear regression analyses.

Total costs for all RBC transfusions and the potential cost savings from eliminating transfusion events with a pre-transfusion hemoglobin value at or above 70 g/L, within 5–10 g/L increments, were estimated. The cost of transfusing a single RBC unit in Alberta was estimated to be $666.10 Canadian dollars (CAD) from a previous activity-based costing study [25]. Given that the volume of a RBC unit can vary between 250 and 350 mL, it was assumed that the volume of a 1 RBC unit was equal to 300 mL in order to estimate the number of RBC units transfused per transfusion event [26]. Costs are reported in 2017 CAD. All data analyses were completed using STATA 13.1 IC.

## Results

### ICU patient characteristics

**Fig. 1:**
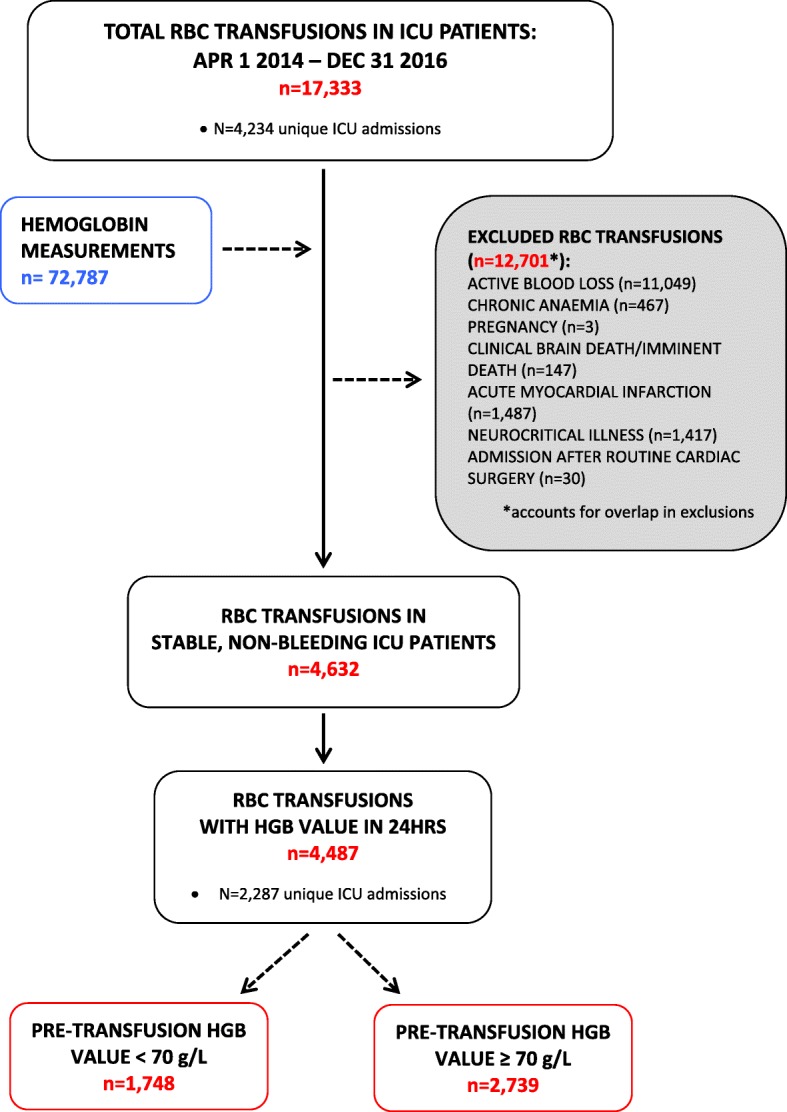
Flow chart of included RBC transfusion events

A total of 2287 ICU admissions, comprised of 2142 unique patients who received at least one RBC transfusion and had a minimum ICU LOS of 24 h, were included (Table 2). The admissions included in the final cohort represented 10.9% of all ICU admissions in the nine participating ICUs during the 33-month study period (2287 out of 4234 admissions). A flow chart of the included RBC transfusion events during these ICU admissions is provided in Fig. 1. Most RBC transfusions were excluded due to evidence of active blood loss or hemorrhage, a primary diagnosis of neurocritical illness, and a primary or secondary diagnosis of acute myocardial infarction.

**Table 2:** Characteristics of ICU patients receiving a RBC transfusion between April 1, 2014 and December 31, 2016

Characteristic	Total frequency
Total included ICU admissions, *n*	2287
Unique patients	2142
Mean age, years (SD)	58.6 (15.5)
< 55 years, *n* (%)	810 (35.4)
55–64 years, *n* (%)	594 (26.0)
65–74 years, *n* (%)	537 (23.5)
≥ 75 years, *n* (%)	346 (15.1)
Gender, *n* (%)	
Male	1240 (54.2)
Female	1047 (45.8)
Mean APACHE II score (SD)	24.4 (8.0)
APACHE II score ≤ 20, *n* (%)	762 (33.3)
APACHE II score > 20, *n* (%)	1525 (66.7)
Mean SOFA score (SD)	9.1 (4.1)
SOFA score < 10, *n* (%)	1259 (55.1)
SOFA score ≥ 10, *n* (%)	1028 (44.9)
Location transferred from, *n* (%)	
Emergency department	672 (29.4)
Operating or recovery room	447 (19.6)
Other ICU	43 (1.9)
Other	1125 (49.1)
ICU admit diagnostic category (or surgery for), *n* (%)	
Infection	612 (26.8)
Gastrointestinal	350 (15.3)
Gastrointestinal bleeding	62 (2.7)
Sepsis	257 (11.2)
Cardiovascular	232 (10.1)
Hepatic-renal	164 (7.2)
Respiratory	135 (5.9)
Trauma	128 (5.6)
Pancreatic	56 (2.5)
Cancer	48 (2.1)
Orthopedic	32 (1.4)
Drug overdose	19 (0.8)
Other	192 (8.4)
Charlson comorbidity index, *n* (%)	
0	642 (28.1)
1	540 (23.6)
≥ 2	1105 (48.3)
Mechanical ventilation, *n* (%)	1922 (84.0)
Length of stay	
Median ICU, days (IQR)	8.1 (11.2)
Mean ICU, days (SD)	12.4 (14.3)
Median hospital, days (IQR)	25.5 (37.5)
Mean hospital, days (SD)	40.7 (45.5)
Readmission, *n* (%)	
ICU within 72 h	61 (2.7)
Hospital within 30 days	330 (14.4)
Hospital within 60 days	497 (21.7)
Mortality, *n* (%)	
ICU mortality	430 (18.8)
Hospital mortality	664 (29.0)

The characteristics of the included cohort are summarized in Table 2. Most patients were under the age of 65 (mean age 58.6 ± 15.5 years) and male (54.2%). The primary ICU admission diagnosis for this cohort was infection (26.8%), and the mean APACHE II and SOFA scores were 24.4 ± 8.0 and 9.1 ± 4.1, respectively. Almost half of the ICU admissions were transferred from the emergency department (29.4%) or the operating or recovery room (19.6%), and the median ICU LOS was just over a week (8.1 days, IQR 11.2). Readmission to the ICU within 72 h was less than 3% (*n* = 61), and ICU mortality was 18.8% (*n* = 430).

### RBC transfusion events and pre-transfusion hemoglobin values

The frequencies of RBC transfusion events are provided in Table 3. A total of 4632 RBC transfusions were identified among the included ICU admissions; this represented approximately 26.7% of all RBC transfusions during the study period (Fig. 1). Assuming that 1 RBC unit was equal to 300 mL [26], approximately 1 RBC unit was transfused per transfusion event, with two transfusion events occurring per ICU admission. With regards to the timing of the RBC transfusion orders, approximately 46% occurred during overnight shifts (i.e., between 19:00 and 07:00) (Table 3).

**Table 3:** Summary of RBC transfusions and pre-transfusion hemoglobin values

Variable	All ICUs
Total RBC transfusion events, *n*	4632
Mean number of transfusions per admission	2.0
Total volume of RBCs transfused, mL	1,356,695
Mean number of RBC units per transfusion	1.0
Overnight transfusions (between 19:00 and 7:00), *n* (%)	2120 (45.8)
Transfusions with hemoglobin measurements ≤ 24 h, *n* (%)	4487 (96.9)
Mean number of hours pre-transfusion (SD)	6.3 (5.3)
Mean pre-transfusion hemoglobin value, g/L (SD)	73.4 (9.2)

**Fig. 2:**
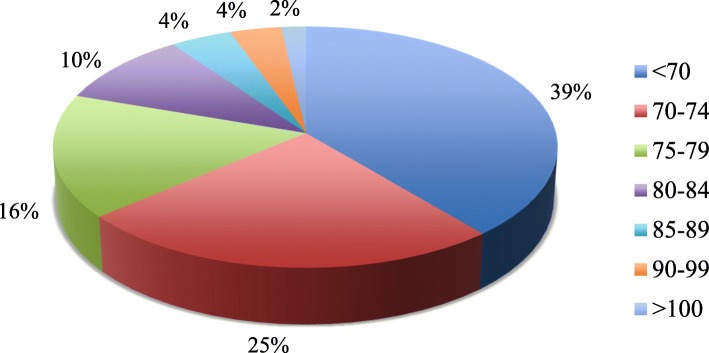
Distribution of pre-transfusion hemoglobin values in grams per liter ranges

**Fig. 3:**
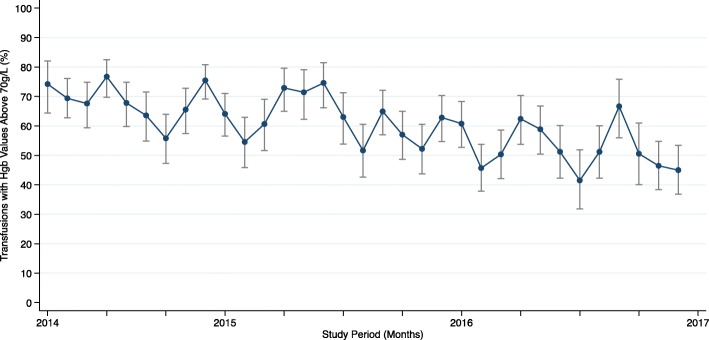
Monthly percentages of RBC transfusion with pre-transfusion hemoglobin values of 70 g/L or more

Pre-transfusion hemoglobin values were identified for 4487 of the RBC transfusions (Table 3), and the mean pre-transfusion hemoglobin value was 73.4 ± 9.2 g/L. The distribution of pre-transfusion hemoglobin values within specific ranges is also depicted in Fig. 2. On average, 61% of the included RBC transfusions had a pre-transfusion hemoglobin value greater than or equal to 70 g/L (*n* = 2739), and 19.7% (*n* = 885) had a pre-transfusion hemoglobin value greater than or equal to 80 g/L (Fig. 2; Table 3). The patient characteristics for each of the RBC transfusion events, stratified in three pre-transfusion hemoglobin groups (less than 70 g/L, between 70 and 79 g/L, greater than or equal to 80 g/L) are also provided in Additional file 2. The monthly proportions of RBC transfusions with hemoglobin values greater than or equal to 70 g/L over the study period are presented in Fig. 3. Simple linear regression revealed that there was a significant decrease in the proportion of these transfusions over the 33 months (*β*1 − 0.706; *p* < 0.0001) (Additional file 3).

### Factors associated with pre-transfusion hemoglobin value

Several factors associated with a pre-transfusion hemoglobin level greater than or equal to 70 g/L were identified through multiple logistic regression (Table 4). Specifically, males (OR 1.20, 95% CI 1.06–1.35), over 75 years of age (OR 1.45, 95% CI 1.16–1.80), SOFA score greater than or equal to 10 (OR 1.18, 95% CI 1.02–1.37), admitted to the ICU from an operating or recovery room (OR 1.47, 95% CI 1.21–1.79), and admitted to the ICU due to gastrointestinal bleeding (OR 2.59, 95% CI 1.50–4.46) or trauma (OR 1.88, 94% CI 1.31–2.69) were all associated with increased odds of having a RBC transfusion with a pre-transfusion hemoglobin value greater than or equal to 70 g/L. In contrast, the odds were significantly decreased if the RBC transfusion occurred overnight (i.e., between 19:00 and 7:00) (OR 0.69, 95% CI 0.61–0.78) (Table 4).

**Table 4:** Factors associated with a pre-transfusion hemoglobin value of 70 g/L or more

	Unadjusted OR		Adjusted OR	
Covariates	OR	95% CI	OR	95% CI
Male	1.21	1.07–1.37	1.20	1.06–1.35
Age category				
< 55	Reference		Reference	
55–64	1.04	0.90–1.22	1.04	0.89–1.22
65–74	1.10	0.94–1.29	1.08	0.91–1.28
≥ 75	1.48	1.21–1.82	1.45	1.16–1.80
APACHE II score > 20	1.05	0.92–1.20	1.05	0.89–1.23
SOFA score ≥ 10	1.14	1.01–1.28	1.18	1.02–1.37
Location before ICU admission				
Other	Reference		Reference	
Emergency department	1.11	0.96–1.27	1.09	0.95–1.26
Operating or recovery room	1.59	1.33–1.89	1.47	1.21–1.79
Other ICU	1.13	0.76–1.66	1.14	0.76–1.70
ICU admit diagnostic category				
Other	Reference		Reference	
Infection	0.83	0.66–1.04	0.79	0.62–1.00
Gastrointestinal	1.37	1.05–1.78	1.17	0.89–1.54
Gastrointestinal bleeding	2.43	1.42–4.15	2.59	1.50–4.46
Sepsis	1.19	0.91–1.57	1.17	0.88–1.56
Cardiovascular	1.38	1.04–1.83	1.31	0.98–1.75
Hepatic-renal	0.88	0.65–1.19	0.84	0.62–1.15
Respiratory	0.74	0.53–1.03	0.75	0.53–1.05
Trauma	2.01	1.42–2.84	1.88	1.31–2.69
Pancreatic	0.93	0.64–1.36	0.95	0.65–1.40
Cancer	0.92	0.53–1.59	0.73	0.42–1.29
Orthopedic	1.51	0.78–2.93	1.31	0.66–2.57
Drug overdose	1.54	0.65–3.63	1.63	0.69–3.89
Charlson comorbidity index				
0	Reference		Reference	
1	0.98	0.83–1.16	1.00	0.84–1.19
≥ 2	1.06	0.92–1.23	1.07	0.92–1.25
Mechanical ventilation	1.06	0.87–1.29	1.06	0.86–1.31
Transfused overnight (between 19:00 and 7:00)	0.71	0.63–0.80	0.69	0.61–0.78

### Impact of pre-transfusion hemoglobin values on length of stay and mortality

Having at least one transfusion with a hemoglobin value at or above 70 g/L was associated with a decrease in ICU LOS (*β*_1_ − 1.63; *p* = 0.029) and hospital LOS (*β*_1_ − 5.36; *p* = 0.002) (Table 5). The association between pre-transfusion hemoglobin value and mortality differed between the ICU and hospital. The adjusted odds of ICU mortality were increased (OR 1.23, 95% CI 1.06–1.44) if a transfusion event was associated with a pre-transfusion hemoglobin of 70 g/L or more; there was no association with hospital mortality (OR 1.14, 95% CI 0.99–1.31) (Table 6).

**Table 5:** Associations between RBC transfusions with a hemoglobin value of 70 g/L or more and ICU and hospital length of stay

Outcome	*β*_1_	SE	95% CI	*r* ^2^	*p* value
ICU LOS	− 1.63	0.75	− 3.09 to − 0.17	0.19	0.029
Hospital LOS	− 5.36	1.76	− 8.81 to − 1.91	0.09	0.002

**Table 6:** Associations between RBC transfusions with a hemoglobin value of 70 g/L or more and ICU and hospital mortality

	Unadjusted OR		Adjusted OR	
Outcome	OR	95% CI	OR	95% CI
ICU mortality	1.20	1.04 to 1.38	1.23	1.06 to 1.44
Hospital mortality	1.14	1.00 to 1.29	1.14	0.99 to 1.31

### Estimated costs of RBC transfusions

The estimated costs of included RBC transfusion events and potential healthcare cost savings from eliminating transfusions with a hemoglobin level of 70 g/L or more, presented within 5–10 g/L increments, are summarized in Table 7. Assuming 1 RBC unit was transfused per event, the total estimated cost of 4487 RBC units (i.e., transfusions with an identified hemoglobin value) was approximately $2.99M CAD. If the 79 RBC transfusion events with a hemoglobin value at or above 100 g/L were eliminated, the estimated cost savings from those transfusion events alone would be $52,622 CAD. Further, if all 2739 transfusions with a hemoglobin value at or above 70 g/L were avoided, the estimated cost savings would increase to approximately $1.82M CAD.

**Table 7:** Estimated costs of included RBC transfusion events between April 1, 2014 and December 31, 2016

Variable	Frequency (*n*)	Total Costs^1^ ($ CAD)
RBC transfusion events with pre-transfusion hemoglobin values	4487	2.99M
Cost savings from eliminating transfusions with:		
Pre-transfusion hemoglobin value ≥ 100 g/L	79	52,622
Pre-transfusion hemoglobin value ≥ 90 g/L	244	162,528
Pre-transfusion hemoglobin value ≥ 85 g/L	441	293,750
Pre-transfusion hemoglobin value ≥ 80 g/L	885	589,499
Pre-transfusion hemoglobin value ≥ 75 g/L	1626	1.08 M
Pre-transfusion hemoglobin value ≥ 70 g/L	2739	1.82 M

^1^Estimates assume that the cost of a RBC unit is $666.10; all costs are reported in 2017 CAD

## Discussion

In this multi-center, retrospective observational study, the RBC transfusion practices in nine adult medical-surgical ICUs were examined. We identified a total of 4632 unique RBC transfusions events in 2287 mixed medical and surgical ICU admissions. Patients within this cohort received approximately two transfusions per ICU admission, with 1 RBC unit transfused per event, and had a mean pre-transfusion hemoglobin level of 73.4 g/L. Among 4487 RBC transfusion events, 61% were associated with a pre-transfusion hemoglobin value at or above 70 g/L. Over the 33-month study period, this was estimated to cost over $1.8M CAD to the healthcare system. Factors such as being male, 75 years of age or older, admission to the ICU from the operating room, and an ICU admission diagnosis of gastrointestinal bleeding or trauma were positively associated with RBC transfusions events with a hemoglobin value greater or equal to 70 g/L. Moreover, having a pre-transfusion hemoglobin value at or above 70 g/L was also associated with an increase in ICU mortality. There was no impact on overall hospital mortality.

We specifically sought to examine RBC transfusions among critically ill patients for whom a restrictive transfusion strategy is supported by the most current, high-quality evidence [16–21]. In doing so, we acknowledged that a restrictive pre-transfusion hemoglobin threshold will not apply in all clinical situations. For instance, in some hemorrhagic or ischemic events, clinical judgment should subvert laboratory value-based thresholds. To account for such reasonable clinical exclusions, we excluded from analysis a considerable number of patients (i.e., greater than 50% of ICU admissions with a RBC transfusion event) for whom a restrictive transfusion strategy has not been proven safe, nor superior, to a liberal transfusion strategy (e.g., chronic anemia, active blood loss, acute coronary syndrome, myocardial infarction, and neurological or traumatic brain injury) [21]. With our final cohort, we, therefore, aimed to decrease the potential for misclassifying a RBC transfusion event—for which a restrictive transfusion approach would not be appropriate—and allowed a conservative evaluation of RBC transfusion practices in the ICU.

Reduced exposure to RBCs, through the adoption of a restrictive pre-transfusion hemoglobin threshold (i.e., less than 70 g/L), has been increasingly recognized in transfusion guidelines as the best practice for most stable, non-bleeding adult patients in the ICU [7, 15, 27, 28]. Within our cohort, we found that over half of all examined RBC transfusions may have occurred outside of the recommended best practice. While these findings suggest potential over-transfusion in the ICU, there have been minor improvements in transfusion practices over time. The reason for this is unknown but is likely the result of increasing awareness and attention to the subject. We also found that over 40% of RBC transfusions examined in our study were associated with a pre-transfusion hemoglobin value between 70 g/L and 79 g/L. Visual inspection of the stratified patient characteristics (Additional file 2) did not suggest differences between transfusions associated with a hemoglobin value below 70 g/L and 70–79 g/L. Interestingly, previous qualitative studies that have examined factors influencing physician transfusion behaviors found the greatest uncertainty among physicians when deciding whether to transfuse patients with a hemoglobin level within this borderline range [29, 30]. Such uncertainty may similarly be reflected in our findings, and targeted efforts to better inform physician decision-making for transfusing such patients may be warranted.

Similar studies examining the appropriateness of RBC transfusion practices have been described in the literature. Previous retrospective audits, for example, have primarily focused on characterizing mean pre-transfusion hemoglobin levels and found them to range substantially between 71 g/L and 91 g/L for most non-hemorrhagic, ICU patient populations [31–34]. One of the larger studies conducted in the USA, a longitudinal analysis RBC transfusion practice between 1997 and 2007, reported a similar mean pre-transfusion hemoglobin level to our patient cohort after their 10 year follow-up period (significantly decreasing from 79 ± 1.3 to 73 ± 1.3 g/L) [32]. In addition, Netzer et al. [32] observed a significant decrease in the proportion of patients who were transfused at a hemoglobin level of less than 70 g/L. In contrast, studies that have examined average transfusion volumes have reported higher numbers of RBC units per transfusion event compared to our study results, ranging from 2 to 4.3 RBC units [32, 35]. While it is difficult to reconcile specific reasons for observed differences between these previous studies and our present findings, some differences are likely attributable to variations in the patient case-mix, ICU structure or culture, or even the time since the publication (and acceptance) of seminal and relevant literature and guidelines.

We conducted additional analytical investigations that represent novel contributions to the existing literature and offer considerations for policy and practice, as well as future research. The costing analysis, for instance, enabled the valuation of not only RBC transfusions across the ICUs, but also of the opportunity cost of potentially inappropriate practices over the 33-month study period. Presenting such costing outcomes provides another perspective for clinical experts to reflect on as the stewards of healthcare resources. This information can also help healthcare system decision-makers implement interventions whose implementation costs could still yield a reasonable return on investment and, as such, improve the overall efficiency of care delivered.

We also identified several factors associated with RBC transfusions with a hemoglobin level above 70 g/L. In particular, ICU admission diagnoses of gastrointestinal bleeding and trauma were associated with at least a 30–50% increase in the odds of these transfusions. Despite the evidence supporting the use of a restrictive RBC transfusion strategy for both of these patient groups [21], as well as our exclusion of transfusions among patients with active bleeding, the propensity for hemorrhagic outcomes with such conditions may account for the increased odds in the observed cases [36, 37]. Due to the overlap in 95% confidence intervals across point estimates, we did not find any one (or few) factor(s) that markedly increased the odds of transfusion over other factors. This suggests that there may be several drivers, both characterized and uncharacterized (e.g., level of physician experience, cultural influences) in our present work that act in concert and underlie the observed proportion of overuse.

In addition, the downstream impacts of pre-transfusion hemoglobin values in our transfused patient cohort differed by ICU and hospital. With respect to mortality, we found that the odds in the ICU were increased for transfused patients with a pre-transfusion hemoglobin of 70 g/L or more, but there was no difference in hospital deaths. Previous meta-analyses of RCTs comparing restrictive versus liberal hemoglobin thresholds found that there was no difference in ICU mortality between groups, yet the risk ratio for hospital mortality was lower among those randomized to the restrictive threshold [21]. While these differences in mortality outcomes may be due to unmeasured confounding in our observational data, our present findings still do not indicate an increased risk of harm for transfused patients with a lower pre-transfusion hemoglobin value (i.e., below 70 g/L).

### Study limitations

There are limitations to the present study worth noting. While we were able to capitalize on population-based administrative and clinical data sources, we may have excluded possible confounders and/or introduced other sources of bias due to the retrospective observational design of the study. Moreover, we did not have adequate recording in the data of the exact reason for a given transfusion across all ICUs. Therefore, patients may have been misclassified and actually had hemorrhage or ischemia. While our conservative inclusion criteria were applied in attempt to mitigate such misclassification, supplementing secondary data sources with medical record audits may additionally aid in overcoming misclassification.

Given the de-identified nature of the data, we were unable to examine the association of factors such as hospital type (i.e., teaching hospital, community hospital) and level of physician experience in our logistic regression analyses. We were also unable to identify the consequences directly associated with a potentially inappropriate transfusion (i.e., transfusion-related complications) due to the retrospective design of our study. Lastly, the present analysis focused on RBC transfusion events in medical-surgical ICUs in one Canadian province; the generalizability of our findings to other Canadian and/or international critical care contexts is unclear. Future studies designed with more detailed prospective data collection and with inter-provincial or international cohort comparisons could, therefore, expand understanding in these areas.

## Conclusions

The present study examined the utilization and costs of RBC transfusion practices within nine medical-surgical ICUs and identified that more than half are still provided above guideline-recommended pre-transfusion hemoglobin thresholds. There is still a large opportunity for improvement in RBC transfusion practices from a patient safety, quality, and financial perspective. Future work to fully characterize the rationale for transfusion decisions as well as understand the factors that underlie the behavior, whether at the environmental or individual physician level, may aid in developing targeted interventions to eliminate overuse [29, 30]. Importantly, the lessons learned from the present study may also help to inform larger-scale blood management programs beyond critical care [38]. With increasing pressures faced by healthcare systems globally to improve quality of care in light of scarce resources, such efforts to address medical overuse are of increasing necessity.

## Additional files

Additional file 1: Operational definitions for exclusion criteria. (DOCX 31 kb)

Additional file 2: Characteristics of ICU patients associated with included RBC transfusions, stratified by pre-transfusion hemoglobin values. (DOCX 93 kb)

Additional file 3: Association between study month and percentage of RBC transfusions with a hemoglobin value of 70 g/L or more. (DOCX 22 kb)

## Abbreviations

APACHE: Acute Physiology and Chronic Health Evaluation
CAD: Canadian dollars
CI: Confidence interval
CIHI: Canadian Institute for Health Information
DAD: Discharge Abstract Database
ICU: Intensive care unit
IQR: Interquartile range
LOS: Length of stay
OR: Odds ratio
RBC: Red blood cell
SD: Standard deviation
SOFA: Sequential Organ Failure Assessment
