# Supplementary material for: A retrospective observational analysis of red blood cell transfusion practices in stable, non-bleeding adult patients admitted to nine medical-surgical intensive care units
Source: J Intensive Care. 2019 Apr 4;7:19. doi: 10.1186/s40560-019-0375-3 (PMC6449900; doi:10.1186/s40560-019-0375-3)
Supplement: Supplementary file 1 — Operational definitions for exclusion criteria. (DOCX 31 kb) [file 40560_2019_375_MOESM1_ESM.docx]

**Additional file 1**. Operational Definitions for Exclusion Criteria

| **Exclusion Criteria** | **Definition(s)** | **eCritical database** | | **DAD database** | |
| --- | --- | --- | --- | --- | --- |
|  |  | **Variables** | **Values/Process** | **Variable Name** | **ICD-10-CA Codes** |
| 1. **Not an adult** | Age less than 18 years at time of ICU admission | - Age | - Equal to or greater than 18 |  |  |
| 1. **Active Blood Loss Prior to Transfusion** | Decrease in Hgb of 30 g/L 12 hrs before the index transfusion | - Date and time of hemoglobin measurement - Value of hemoglobin measurement - Date and time of RBC transfusion | - Identify all hgb measurements 12 hrs prior to txn event - Flag any drops in hgb greater than or equal to 30 g/L (between any sequential events) |  |  |
|  | 3 units (900mL) of RBC 12 hrs before the index transfusion | - Date and time of RBC transfusion - Volume of RBC transfused | - Identify individual transfusions with volumes greater than or equal to 900mL   AND   - Identify transfusion events in the 12 hrs prior to the index transfusion event - Flag cumulative transfusion volumes greater than or equal to 900mL |  |  |
|  | One or more transfusions 12hrs after the index transfusion | - Date and time of RBC transfusion | - Identify all transfusion events in the 12 hrs after the index transfusion event |  |  |
|  | Other indicators of active blood loss | - Admit ICU diagnosis | - Hemorrhage (for gastrointestinal bleeding GI-see GI system) (for trauma see Trauma) - Hemorrhage, intra/retroperitoneal - Hemorrhage, postpartum (female only) - Hemorrhage/hemoptysis, pulmonary |  |  |
|  |  | - Cardiovascular reasons | - Hypovolemic Hemorrhagic Shock or Non Hemorrhagic Shock |  |  |
|  |  | - Bleeding events | - “Bleeding” indicated if it occurred |  |  |
| 1. **Anemia** | Primary or secondary diagnosis of anemia | - Admit ICU diagnosis | - Anemia | Diagnosis 1-25 | - D50.0 (Iron deficiency anaemia secondary to blood loss [chronic],   Includes: Posthaemorrhagic anaemia [chronic]) |
| 1. **Pregnancy** | Pregnant state during in-hospital stay | - Admit ICU diagnosis | - Pregnancy | Diagnosis 1-25 | - Z33 (Pregnant state incidental) |
| 1. **Brain death or imminent death** | Indication of clinical brain death in ICU or death within 24hrs of ICU admission | - Clinical brain death | - “YES” if indicated | Diagnosis 1-25 | - G93.81 (Neurologically determined death) |
|  |  | - Time of death | - Calculate time difference between admission to ICU and death - Flag death less than 24 hrs in ICU |  |  |
| 1. **Acute myocardial infarction (AMI)** | Primary or secondary diagnosis of AMI | - Admit ICU diagnosis | - Infarction, acute myocardial (MI) | Diagnosis 1-25 | - I21.xx (Acute myocardial infarction) - I22.xx (Subsequent myocardial infarction) |
| 1. **Neurocritical illness** | Critically ill patients with neurological injury or condition of the brain requiring intensive care treatment | - Admit ICU diagnosis | - Head (CNS) only trauma - Head (CNS) only trauma, surgery for - Head/abdomen trauma - Head/abdomen trauma, surgery for - Head/chest trauma - Head/extremity trauma - Head/extremity trauma, surgery for - Head/face trauma - Head/face trauma, surgery for - Head/multiple trauma - Head/multiple trauma, surgery for - Head/pelvis trauma - Head/pelvis trauma, surgery for - Head/spinal trauma - Head/spinal trauma, surgery for - Hematoma, epidural, surgery for - Hematoma, subdural - Hematoma, subdural, surgery for - CVA, cerebrovascular accident/stroke - Subarachnoid hemorrhage/arteriovenous malformation - Subarachnoid hemorrhage/intracranial aneurysm - Subarachnoid hemorrhage/intracranial aneurysm, surgery for - Hemorrhage/hematoma, intracranial - Hemorrhage/hematoma-intracranial, surgery for - Seizures (primary-no structural brain disease) - Seizures-intractable, surgery for - Encephalitis - Encephalopathies (excluding hepatic) - Encephalopathy, hepatic - Neurological medical, other - Neurological surgery, other - Abscess, neurologic - Nontraumatic coma due to anoxia/ischemia - Neoplasm, neurologic - Neoplasm-cranial, surgery for (excluding transphenoidal) - Abscess, neurologic - Cranioplasty and complications from previous craniotomies | Diagnosis 1 | - S06.xx (Intracranial injury) - S07.xx (Crushing injury of head) - I60.xx (Subarachnoid haemorrhage) - I61.xx (Intracerebral haemorrhage) - I62.xx (Other nontraumatic intracranial haemorrhage) - I63.xx (Cerebral infarction) - I64.xx (Stroke, not specified as haemorrhage or infarction) - G40.xx (Epilepsy) - G41.x (Status epilepticus) - R56.xx (Convulsions, not elsewhere classified) - G04.x (Encephalitis, myelitis and encephalomyelitis) - G05.x (Encephalitis, myelitis and encephalomyelitis in diseases classified elsewhere) - G06.0 (Intracranial abscess and granuloma) - G91.x (Hydrocephalus) - G92 (Toxic encephalopathy) - C71.x (Malignant neoplasm of brain) - G93.xx (Other disorders of brain) - G94.x (Other disorders of brain in diseases classified elsewhere) |
| 1. **ICU admission after routine cardiac surgery** | ICU admission of post-operative cardiac patients | - Admit ICU diagnosis | - Cardiovascular surgery, other - CABG alone, coronary artery bypass grafting |  |  |
