# Supplementary material for: A retrospective observational analysis of red blood cell transfusion practices in stable, non-bleeding adult patients admitted to nine medical-surgical intensive care units
Source: J Intensive Care. 2019 Apr 4;7:19. doi: 10.1186/s40560-019-0375-3 (PMC6449900; doi:10.1186/s40560-019-0375-3)
Supplement: Supplementary file 2 — Characteristics of ICU patients associated with included RBC transfusions, stratified by pre-transfusion hemoglobin values. (DOCX 93 kb) [file 40560_2019_375_MOESM2_ESM.docx]

**Additional file 2.** Characteristics of ICU Patients Associated with included RBC Transfusions, Stratified by Pre-transfusion Hemoglobin Values

|  | **Pre-Transfusion Hemoglobin Category** | | |
| --- | --- | --- | --- |
| **Characteristic** | **< 70 g/L** | **70-79 g/L** | **≥ 80 g/L** |
| **Transfusions with Hemoglobin Measurements ≤ 24 hrs,**  **n (%)** | 1,748 (39.0) | 1,854 (41.3) | 885 (19.7) |
| **Mean age, years (SD)** | 56.1 (15.2) | 57.9 (14.4) | 59.0 (15.6) |
| < 55 years, n (%) | 709 (40.6) | 710 (38.3) | 317 (35.8) |
| 55 – 64 years, n (%) | 479 (27.4) | 516 (27.8) | 210 (23.7) |
| 65 – 74 years, n (%) | 392 (22.4) | 410 (22.1) | 215 (24.3) |
| ≥ 75 years, n (%) | 168 (9.6) | 218 (11.8) | 143 (16.2) |
| **Gender, n (%)** |  |  |  |
| Male | 897 (51.3) | 1,025 (55.3) | 513 (58.0) |
| Female | 851 (48.7) | 829 (44.7) | 372 (42.0) |
| **Mean APACHE II Score (SD)** | 24.9 (7.5) | 25.2 (7.5) | 25.5 (8.7) |
| APACHE II Score ≤ 20, n (%) | 496 (28.4) | 494 (26.7) | 255 (28.8) |
| APACHE II Score > 20, n (%) | 1,252 (71.6) | 1,360 (73.4) | 630 (71.2) |
| **Mean SOFA Score (SD)** | 9.6 (4.0) | 10.1 (4.0) | 10.1 (4.3) |
| SOFA Score < 10, n (%) | 828 (47.4) | 810 (43.7) | 401 (45.3) |
| SOFA Score ≥ 10, n (%) | 920 (52.6) | 1,044 (56.3) | 484 (54.7) |
| **Location Transferred From, n (%)** |  |  |  |
| Emergency Department | 519 (29.7) | 529 (28.5) | 278 (31.4) |
| Operating or Recovery Room | 239 (13.7) | 340 (18.3) | 193 (21.8) |
| Other ICU | 43 (2.5) | 55 (3.0) | 13 (1.5) |
| Other | 947 (54.2) | 930 (50.2) | 401 (45.3) |
| **ICU Admit Diagnostic Category (or surgery for), n (%)** |  |  |  |
| Infection | 594 (34.0) | 528 (28.5) | 191 (21.6) |
| Gastrointestinal | 214 (12.2) | 272 (14.7) | 157 (17.7) |
| Gastrointestinal Bleeding | 20 (1.1) | 36 (1.9) | 35 (4.0) |
| Sepsis | 185 (10.6) | 214 (11.5) | 109 (12.3) |
| Cardiovascular | 151 (8.6) | 176 (9.5) | 128 (14.5) |
| Hepatic-Renal | 141 (8.1) | 142 (7.7) | 40 (4.5) |
| Respiratory | 110 (6.3) | 89 (4.8) | 30 (3.4) |
| Trauma | 66 (3.8) | 117 (6.3) | 77 (8.7) |
| Pancreatic | 68 (3.9) | 71 (3.8) | 22 (2.5) |
| Cancer | 26 (1.5) | 24 (1.3) | 11 (1.2) |
| Orthopaedic | 14 (0.8) | 18 (1.0) | 13 (1.5) |
| Drug overdose | * | 13 (1.0) | * |
| Other | 151 (8.6) | 154 (8.3) | 67 (7.6) |
| **Charlson Comorbidity Index, n (%)** |  |  |  |
| 0 | 506 (29.0) | 510 (27.5) | 263 (29.7) |
| 1 | 405 (23.2) | 418 (22.6) | 188 (21.2) |
| ≥ 2 | 837 (47.9) | 926 (49.6) | 434 (49.0) |
| **Mechanical Ventilation, n (%)** | 1,565 (89.5) | 1,668 (90.0) | 798 (90.2) |
| **Length of Stay** |  |  |  |
| Median ICU, days (IQR) | 14.9 (22.1) | 13.6 (20.9) | 10.7 (16.9) |
| Mean ICU, days (SD) | 23.0 (24.9) | 23.0 (31.3) | 19.2 (28.5) |
| Median Hospital, days (IQR) | 36.9 (49.5) | 34.7 (48.5) | 28.5 (50.4) |
| Mean Hospital, days (SD) | 55.7 (60.4) | 53.9 (56.7) | 47.4 (52.6) |
| **Readmission, n (%)** |  |  |  |
| ICU within 72 hrs | 41 (2.4) | 62 (3.3) | 35 (4.0) |
| Hospital within 30d | 227 (13.0) | 284 (15.3) | 105 (11.9) |
| Hospital within 60d | 359 (20.5) | 399 (21.5) | 163 (18.4) |
| **Mortality, n (%)** |  |  |  |
| ICU mortality | 394 (22.5) | 442 (23.8) | 267 (30.2) |
| Hospital mortality | 576 (33.0) | 629 (33.9) | 352 (39.8) |

*cells with counts less than 10 were masked
