# Supplementary material for: A retrospective observational analysis of red blood cell transfusion practices in stable, non-bleeding adult patients admitted to nine medical-surgical intensive care units
Source: J Intensive Care. 2019 Apr 4;7:19. doi: 10.1186/s40560-019-0375-3 (PMC6449900; doi:10.1186/s40560-019-0375-3)
Supplement: Supplementary file 3 — Association between study month and percentage of RBC transfusions with a hemoglobin value of 70 g/L or more. (DOCX 22 kb) [file 40560_2019_375_MOESM3_ESM.docx]

**Additional file 3.** Association between Study Month and Percentage of RBC Transfusions with Hemoglobin Value of 70 g/L or more

| **β_1_*** | **SE** | **95% CI** | **r^2^** | **p-value** |
| --- | --- | --- | --- | --- |
| -0.706 | 0.130 | -0.972-0.441 | 0.4871 | <0.0001 |

*change in proportion per month of study period
